# Alexithymia in Anorexia Nervosa, More State Than Trait? A Multi‐Dimensional Assessment of Emotion Processing in Patients With Anorexia Nervosa, Recovered Patients and Healthy Controls

**DOI:** 10.1002/erv.70125

**Published:** 2026-05-19

**Authors:** E. Wezenberg, S. K. Peters, A. A. van Elburg, S. H. W. Mares

**Affiliations:** ^1^ Department of Eating Disorders (Amarum) GGNet Mental Health Warnsveld the Netherlands; ^2^ Radboud University Nijmegen Behavioural Science Institute Nijmegen the Netherlands; ^3^ Department of Clinical Psychology Utrecht University Utrecht the Netherlands; ^4^ Co‐Eur Organisation for Eating Disorders Utrecht the Netherlands

**Keywords:** alexithymia, anorexia nervosa, behavioural, physiological, recovered patients

## Abstract

**Objective:**

Alexithymia is implicated in both development and maintenance of Anorexia Nervosa (AN). It is unclear what defines alexithymia in AN. In this study, emotion processing related to alexithymia was examined.

**Method:**

40 patients with AN, 43 recovered (RC) patients and 35 matched healthy controls (HC) filled out questionnaires on eating disorder pathology (EDE‐Q), depression (CES‐D), alexithymia (TAS‐20), and emotion regulation (DERS). BMI was obtained. Behavioural (naming emotions, valence and intensity ratings) and physiological responses (heart rate and skin conductance) to emotional stimuli were assessed. Data were analysed for group differences, and controlled for effects of depression.

**Results:**

Although high levels of negative affect, alexithymia and emotion regulation problems were found in self‐report questionnaires, the AN group was able to identify and name simple explicit emotions, and showed only subtle signs of reduced physiological responsiveness when viewing the emotion pictures. The RC group only showed differences to the healthy control group in self‐assessed alexithymia.

**Conclusions:**

The present results give more evidence of alexithymia as a state than a trait feature in AN. Longitudinal studies are advised to gain more insight into how eating disorder behaviours versus alexithymia and emotion regulation change during the recovery process of AN.

## Highlights

- The current comparison of multiple groups with an extensive mix of measures (self‐reports, behavioural and physiological) provides a differentiated insight in emotion processing in AN. The AN group reports more problems in emotion recognition and expression than how they perform.
- The recovered group hardly differs from healthy controls in most clinical, behavioural or physiological measures of eating disorder psychopathology and emotion processing.
- Alexithymia in AN appears to be more state than trait.

## 1 Introduction

A considerable body of research has demonstrated that individuals with anorexia nervosa (AN) frequently experience difficulties in identifying and describing emotions and have an externally oriented thinking style, which is referred to as alexithymia (Westwood et al. 2017). Next to alexithymia, emotion regulation difficulties have been established as core features of AN, and both play an important role in the development and maintenance of the disease (Leppanen et al. 2022; Mallorquí‐Bagué et al. 2018; Wong et al. 2025). Patients with AN show low rates of recovery; the disorder often becomes chronic (Eddy et al. 2017) and underlying deficits in emotion processing related to alexithymia and emotion regulation are thought to play a role, next to state related features like starvation (Gramaglia et al. 2020; Lavender et al. 2015; Oldershaw et al. 2015; Zeiler et al. 2025).

As the ability to identify and understand one's emotions is essential in choosing the right emotion regulation strategy, this leads to more maladaptive choices in individuals with high alexithymia levels (Preece et al. 2023). In the original model of alexithymia (Sifneos, 1972), it was assumed that alexithymic individuals have an absence or reduced ability to notice their emotions due to an impairment in the ability to verbalise emotions, in the presence of an intact physiology underlying emotional experience (Taylor et al. 1997). Alexithymia is seen as a personality‐like, transdiagnostic trait in many different diagnostic classifications next to eating disorders as well (Luminet et al. 2021). Patients with anorexia nervosa differ from the other diagnostic groups in that they experience physical effects from their disease due to starvation, like bradycardia, which raises the question whether reduced heart rate might hamper emotion processing and whether the original alexithymia model is applicable to patients with anorexia nervosa. Does alexithymia in AN mean that these patients don't notice their feeling as much as other people due to different physiological responses to emotions? Or does it mean that they are only limited in their ability to verbalise their internal experiences? This paper will dive deeper into this topic to try to clarify the role of alexithymia, and the related concepts emotion recognition and expression in AN.

First, an overview will be given on existing research findings. Most alexithymia research has been done with the TAS‐20, a well‐known self‐report questionnaire of alexithymia (Westwood et al. 2017). A limitation of self‐report questionnaires however, is that with the inability of participants to verbalise or access one's own emotions, the outcome of self‐report questionnaires might be biased (Donges and Suslow 2017).

Behavioural assessments provide a more objective measure of alexithymia, particularly in emotion recognition and expression. There are only a few behavioural studies that focussed on the recognition and expression of emotions subjects themselves (as most studies focus on the social context). Overall, the behavioural studies suggest diminished emotion recognition and expression in AN (Jänsch et al. 2009; Joos et al. 2009; Davies et al. 2011, 2012), though the current body of behavioural research on this topic remains limited.

Another relevant field is the physiological measurements of emotions. There appears some initial evidence for hypo‐arousal in patients with AN during baseline measures of heart rate (HR), skin conductance (SCR) and electromyography. Palomba et al. 2017; Petretta et al. 1997; Rommel et al. 2015, as well as in studies looking into physiological responses to emotion inducting stimuli (Zonnevylle‐Bender et al. 2005; Liao et al. 2009; Tchanturia et al. 2007; Soussignan et al. 2010, 2011; Bellodi et al. 2013). Although there are also studies that no differences between groups in physiological responding (Soussignan et al. 2010; Yiu et al. 2018). When physiological processing of emotions is reduced in patients with AN, this might hamper emotion recognition and expression, so far the evidence is insufficient to draw strong conclusions.

It is noteworthy that few studies have combined these different methods to investigate emotional recognition and expression in AN patients versus healthy control participants (HC), which will be the focus of the present study. Furthermore, an additional group with recovered patients (RC) would give information on the question whether alexithymia is more a state than a trait associated feature of the illness. So, the aim of the present study is to gain a better understanding of the role of alexithymia in emotion processing in AN by combining self‐report, behavioural and physiological measures in three different groups (patients with AN, HC, and RC). For this purpose, a well‐known research paradigm for measuring both behavioural and physiological responses to emotions was used: The International Affective Picture System (IAPS) from Lang et al. (1999). A new condition was added to this paradigm, in which subjects verbalise their experienced emotions. The aim was to gain a more complete picture of emotion recognition and expression in AN that is thought to be impaired in these patients with high levels of alexithymia.

To conclude, It is hypothesised that patients with AN will show higher scores on self‐report questionnaires of alexithymia and emotion regulation difficulties compared to HC participants, replicating earlier research. The RC group is thought to perform in between the other two groups., based on earlier findings that alexithymia usually significantly improves with weight and clinical recovery, but often not fully to the level of healthy controls and some difficulties in emotion regulation remain (Castro et al. 2021).

Secondly, it is hypothesised that AN patients will have more difficulty in giving verbal emotion expression to emotion stimuli (i.e., less use of emotion words), and will show reduced rating of valence and intensity than HC.

Thirdly, it is hypothesised that patients with AN will show signs of hypo‐arousal and have less baseline physiological response, as well as less physiological response when viewing intense emotion pictures compared to HC, even though earlier research findings were mixed. For the RC group so far, no data exist for behavioural and physiological measures, however if alexithymia is a trait rather than a state feature in the RC group, the RC group is expected to perform more like the AN‐ than the HC‐group.

Depression is a highly prevalent comorbidity in patients with AN (Hambleton et al. 2022). Depression and alexithymia symptoms often co‐occur in AN and these constructs are seen as partially overlapping, but not identical constructs (Torres et al. 2015; Zeiler et al. 2025), therefore depression ratings are added as a covariate to the analyses.

## 2 Methods

### 2.1 Setting and Design

The data collection took place between August 2017 and March 2023 at GGNet Amarum, an expert centre for eating disorders in the Netherlands, and at the Radboud University Nijmegen.

A combined behavioural and physiological paradigm was used: the IAPS (Lang et al. 1999).

A cross‐sectional design was used with partially a between subject and partially a three‐way mixed model design. The between subject condition consisted of three groups: AN, RC and HC. The within subject condition for the picture viewing task had 3 levels: negative‐, neutral‐ and positive stimulus‐type. The within subject condition for assessment of heartbeat pattern had 5 levels (time laps).

### 2.2 Participants

The study sample consisted of 118 participants, of which 40 patients with AN, 43 RC and 35 HC participants. Patients with AN were recruited from the inpatient and outpatient clinic for eating disorders, GGNet Amarum in The Netherlands. They were diagnosed by trained clinicians and fulfiled DSM 5TR criteria for AN with a SCID‐5‐CV interview. RC patients were recruited through posters via GGNet Amarum, social media, and through Ixta Noa, an organisation with experts by experience. Individuals were considered recovered when weight was normalised (BMI ≥ 18.5) and eating disorder behaviours like binging, vomiting, and use of laxatives or fasting were absent for at least 3 months according to criteria of Bardone‐Cone et al. (2010). Participants in the HC group were recruited through posters at the Radboud University in Nijmegen (The Netherlands), and through the staff and family members and friends of the staff of GGNet Amarum. All participants were female.

Exclusion criteria for all participants were: age below 18 years, intellectual disability (IQ below 80), not able to read and write Dutch, uncorrected visual disability, substance use disorder, or benzodiazepine use before start of the study. For HC and RC in retrospect EDE‐Q scores of two standard deviations above the mean for HC (Aardoom et al. 2012) was used as exclusion criterium.

### 2.3 Procedure

The study was approved by the ethics committee of the department of social sciences of the Radboud University in Nijmegen (ECSW20171303‐496 and ECSW‐2020‐070). The study was carried out in accordance with the Declaration of Helsinki. Prior to the start of the experiment all subjects gave their written informed consent.

First baseline heart rate was assessed. At the start of the experiment, participants were connected to a BioPac, a signal amplifier. Electrodes for HR were attached to the inside of the left ankle (ground) and the inside of both wrists. SCR electrodes were attached to the middle‐ and index finger of the non‐dominant hand. Electrode paste (Ag/AgCl) was used on the SCR electrodes. The BioPac recording device was connected to a laptop with Acqknowledge data acquisition software. Participants were seated in front of a computer monitor on which pictures were displayed. A 5 min baseline period was obtained before start. During the experimental phase, 30 pictures were presented in a fixed (semi‐random) order. There was a 20–25 s interval period between each presented stimulus to prevent expectations to ensure the integrity of the measurement (Braithwaite et al. 2013). The physiological measurement lasted 30 min, after which the electrodes were removed. Afterwards, participants were shown the same stimuli again in a paper‐and‐pencil task and participants were asked to write down one word for each picture which described the emotion that the picture elicited. Next they were asked to rate all pictures on valence and emotional intensity on a 9‐point Likert scale ranging from 1 (‘extremely negative’ and ‘not intense at all’, respectively) to 9 (‘extremely positive’ and ‘very intense’, respectively).

Finally, participants were asked to fill out the questionnaires (paper‐and‐pencil). In addition, to obtain BMI, height and weight were measured by the researchers.

At the end participants were offered a small gift coupon and travelling cost restitution for participating in the experiment.

### 2.4 Materials

The behavioural experiment: Participants were presented 30 pictures, 10 had positive valence, 10 had negative valence, and 10 had neutral valence. All pictures were retrieved from IAPS (Lang et al. 1999). The IAPS pictures are often and effectively used for emotion elicitation (Branco et al. 2023).

The codes of the pictures that were used are:

Positive: 5780, 1463, 5621, 8170, 5480, 2058, 8031, 8496, 5600, 1710;

Negative: 3170, 3000, 1201, 1111, 6260, 9911, 1525, 2120, 6350, 9570;

Neutral: 7090, 7130, 2840, 7010, 7175, 7710, 7590, 7705, 2222, 2385.

The outcome measures were valence and intensity for the ratings of the pictures and the frequency of emotion words and non‐emotion words in the naming condition.

The Cronbach alpha in the present study was the following for valence: positive—*α* = *0*.*756*, negative—*α* = 0.800 and neutral pictures *α* = 0.648; intensity: positive—*α* = 0.882, negative—*α* = 0.905 and neutral pictures *α* = 0.839; frequency of emotion words: positive—*α* = 0.744, negative—*α* = 0.687 and neutral pictures *α* = 0.496; frequency of non‐emotion words: positive—*α* = 0.637, negative—*α* = 0.711 and neutral pictures *α* = 0.807.

The heart rate (HR) and skin conductance (SCR) of all participants were measured using a BioPac MP150 recording device (BIOPAC). The sampling frequency was 1000 hz. For HR the outcome measure was baseline HR and in addition difference scores were calculated for the inter beat interval (IBI) score before stimulus presentation versus the IBI afterwards for the successive six heartbeats. For SCR the outcome measure was the maximum amplitude within 4 s after stimulus presentation.

The Cronbach alpha in the present study for physiological responses to pictures was the following for heartrate: positive—*α* = *0*.*992*, negative—*α* = 0.994 and neutral pictures *α* = 0.997 and for skin conductance: positive—*α* = *0*.*624*, negative—*α* = 0.901 and neutral pictures *α* = 0.848.

#### 2.4.1 Questionnaires

The Eating Disorder Examination Questionnaire (EDE‐Q) is a self‐report questionnaire that is used to assess eating disorder psychopathology. The EDE‐Q consists of 28 questions that asses restraint, shape concerns, eating concerns, and weight concerns over the previous 28 days (Fairburn and Beglin 2008). The EDE‐Q has high internal consistency, test‐retest reliability and discriminative validity (Aardoom et al. 2012). The Cronbach alpha in the present study was 0.964. The mean score was used in the present study.

The Toronto Alexithymia Scale is a self‐report questionnaire that is used to measure the alexithymia construct as defined by Sifneos (1972), who assumed alexithymia to be a problem in verbally describing emotional experience (Taylor et al. 1997). The TAS‐20 is the most commonly used measurement of alexithymia (Westwood et al. 2017). It consists of 20 questions. The factor structure of the TAS‐20 has been supported (Bagby, Taylor, et al. 1994; Bagby, Parker, et al. 1994). The Cronbach alpha in the present study was 0.815. The total score was used in the present study.

The Difficulties in Emotion Regulation Scale (DERS) is a self‐report questionnaire that is used to measure emotion regulation. The DERS consists of 36 statements for which participants have to rate how strongly they agree on a 5‐point Likert scale. The DERS has good test‐retest reliability, adequate predictive validity and construct validity and high internal consistency (Gratz and Roemer 2004). The Cronbach alpha in the present study was 0.909. The total score was used in the present study.

The CES‐D is a self‐report questionnaire which measures depressive symptoms occurring during the week of assessment. The CES‐D consists of 20 statements for which participants have to rate how often in the last week they have been applicable on a scale from 0 (‘never or almost never’) to 3 (‘most of the time or always’). The CES‐D has good internal consistency and validity (Wu et al. 2016). The Cronbach alpha in the present study was 0.959. The total score was used in the present study.

### 2.5 Data Analysis

Physiological data were pre‐processed using BioPac Student Lab PRO and analysed with the Statistical Package for the Social Sciences (SPSS) Version 25.

Missing data: Some subjects did not complete all of self‐report questionnaires after the physiology measurements (EDEQ 0 missing, TAS‐20 3 AN‐, DERS 2 RC missing, CES‐D 3 AN, 1 RC missing). Due to technical malfunction, physiological data was not recorded for some participants (HR 2 RC missing, SCR 4 RC missing).

After reviewing extreme values (difference more than two standard deviations above (or below) the mean score), one datapoint (SCR amplitude of HC subject) was removed from the analyses to prevent unjust skewing of the results and changing overall outcome.

Group differences on demographic and clinical questionnaires (EDE‐Q, TAS‐20, DERS and CES‐D) were analysed with a one‐way multiple analysis of variance (MANOVA).

To examine difficulty in naming emotions, written responses and ratings on the emotion picture viewing task were assessed.

The emotion words written down by participants in response to the emotion pictures were categorised by three independent raters with a profession in psychiatry into the category: emotion response versus no emotion response (something completely different). The interrater reliability was calculated and substantial: kappa interrater A versus B *κ* = 0.69; A versus C *κ* = 0.75 & B versus C *κ* = 0.77.

The frequency of emotion responses, written emotion words versus no emotion words, was counted for all 30 pictures, resulting in a sum‐score for emotion words and non‐emotion words. To determine group differences in these conditions a repeated measures ANOVA was performed with the sum‐score of emotion versus non‐emotion words as dependent factor, stimulus type as a within subject factor and group as a between subject factor.

To assess group differences in valence and arousal ratings in the emotion picture viewing task, two repeated measures ANOVA's were conducted. One with ‘valence’ and the other with ‘intensity’ as dependent variables, both with stimulus type (positive, negative, neutral) as within subject variable and group as between subject variable.

Group differences in baseline heartrate, and heart rate response were analysed by a GLM repeated measures ANOVA, with group (AN, RC, HC) as between subject variable and stimulus type (negative, neutral and positive) and BPM HR change in time laps (difference from pre‐stim HR on second, third, fourth, fifth, 6th heart beat after stimulus presentation) as within‐subject variables (Bradley et al. 2001).

SCR response was determined by the maximum amplitude the first 4 s after stimulus presentation (Braithwaite et al. 2013; Dawson et al. 2016). Group differences in skin conductance response were analysed by a GLM repeated measures ANOVA, with group (AN, RC, HC) as between subject variable and stimulus type (negative, neutral and positive) as within subject variable.

Results were checked and adjusted for possible sphericity with Greenhouse‐Geisser corrections.

Partial *η* ^2^ was used to report effect sizes for (repeated measures) ANOVA's: small (0.01), medium (0.06) and large (0.14) (Cohen 1992).

To control for possible interfering effect of depression, analyses of covariance was done with CES‐D ratings as covariate.

## 3 Results

### 3.1 Alexithymia and Emotion Regulation in Self‐Report Questionnaires

Table 1 shows the demographic and clinical characteristics. Multivariate statistics (*F* (10, 206) = 31.85, *p* < 0.001) revealed a significant group effects on the clinical measures combined. Subsequent analyses of between‐subject effects are reported in Table 1.

**TABLE 1:** Demographic variables and clinical characteristics for the three groups: Anorexia nervosa (AN), recovered anorexia nervosa (RC) and healthy controls (HC).

	AN (*n* = 40)		RC (*n* = 43)		HC (*n* = 35)				Posthoc contrast
	Mean	sd	Mean	sd	Mean	sd	F (2,107)	*η* ^2^	
Age	29.43	11.57	32.65	10.73	28.26	10.50	1.07	0.19	
Education^a^	5.50	1.06	6.16	0.61	6.49	0.56	6.54^***^	0.19	AN < RC = HC
BMI	16.45	2.01	22.28	3.77	22.03	3.76	32.5^***^	0.38	AN < RC = HC
EDE‐Q	4.21	1.21	0.82	0.92	0.72	0.68	152.4^***^	0.74	AN > RC = HC
CES‐D	40.09	10.13	10.50	9.48	9.17	7.31	132.4^***^	0.71	AN > RC = HC
TAS‐20	64.17	9.98	42.95	9.44	37.30	7.62	85.9^***^	0.62	AN > RC > HC
DERS	129.00	20.44	80.85	19.83	69.71	20.45	85.9^***^	0.62	AN > RC = HC

Abbreviations: BMI = Body Mass Index, CES‐D = Centre for Epidemiologic Studies Depression Scale, DERS = Difficulties in Emotion Regulation Scale, EDE‐Q = Eating Disorder Questionnaire, TAS‐20 = Toronto Alexithymia Scale.

^a^ Verhage‐dutch education coding (Kalesse et al. 2024).

^#^ *p* < 0.1, *p < 0.05, **p < 0.01, ***p < 0.001.

The first hypothesis was that patients with AN would show higher scores on self‐report questionnaires measuring alexithymia and difficulties with emotion regulation than HC participants, with the RC group rating somewhere between the other two groups. The AN group showed very high scores on all clinical measures. As expected, the AN group differed significantly from the RC and HC groups in terms of alexithymia and emotion regulation ratings. As anticipated, the RC alexithymia ratings differed significantly from both the HC and AN groups and were in the middle. However, the RC group's ratings were not in the clinical or subclinical range of alexithymia, unlike the AN group (Westwood et al. 2017). Contrary to the hypothesis, however, the RC group did not differ from the HC group in terms of emotion regulation ratings. Additionally, the RC group showed no differences to the HC group in the other clinical measures: eating disorder pathology and depression, whereas these clinical ratings were significantly elevated in the AN group.

### 3.2 Behavioural Measures: Valence and Arousal Ratings and Use of Emotion and Non‐Emotion Words in Response to Viewing Emotion Pictures

Results of the behavioural measures are found in Table 2.

**TABLE 2:** Descriptives, GLM repeated measures ANOVA and ANCOVA results on behavioural and physiological responses before and after viewing emotion pictures.

	Descriptives							GLM repeated measures ANOVA				ANCOVA CES‐D	
	AN		RC		HC								
	Stimtype	Means	sd	Means	sd	Means	sd	Main effects	df	*F*	*η* ^2^	*F*	*η* ^2^
Behavioural measures													
Valence	Negative	2.24	1.04	2.35	0.67	2.21	0.61	Stimtype	2111	847^***^	0.94	241^***^	0.68
	Neutral	4.52	0.73	4.88	0.62	4.87	0.85	Stimt × group	4220	2.04^#^	0.04	1.75	0.12
	Positive	6.70	0.96	7.02	0.64	7.20	0.66	Group	2111	4.09^*^	0.09	< 1	0.01
Intensity	Negative	5.74	1.74	6.37	1.21	6.22	1.26	Stimtype	2111	282.75^***^	0.72	56.84^***^	0.33
	Neutral	3.18	1.34	3.49	1.14	3.10	1.04	Stimt × group	4220	1.64	0.03	1.17	0.02
	Positive	5.00	1.49	5.16	1.44	5.34	1.19	Group	2.111	1.19	0.02	< 1	0.01
Number of emotion words	Negative	4.35	3.29	4.81	2.59	4.97	2.74	Stimtype	2111	127.99^***^	0.70	25.08^***^	0.18
	Neutral	0.84	1.17	0.81	1.03	1.09	1.25	Stimt × group	4220	< 1	0.01	< 1	0.01
	Positive	2.57	2.71	3.00	1.88	3.31	2.27	Group	2111	< 1	0.02	< 1	0.01
Other non‐emotion words	Negative	1.03	1.64	0.40	1.05	0.29	0.67	Stimtype	2111	20.93^***^	0.16	4.28^*^	0.00
	Neutral	2.35	2.67	1.60	1.99	0.94	1.53	Stimt × group	4220	1.19	0.02	< 1	0.01
	Positive	1.49	1.71	1.26	1.51	0.69	1.11	Group	2112	5.27^**^	0.09	< 1	0.02
Physiological measures													
Baseline heartrate (BPM)		69.17	10.24	71.99	11.21	71.76	8.55	Group	2104	< 1	0.02	1.48	0.03
Heartrate change (IBI)	Descriptives depicted in Figure 1							Stimtype	2230	4.14^*^	0.04	3.60^*^	0.03
								Stimt × group	4230	3.05^*^	0.05	2.71^*^	0.05
								Timecours	4460	< 1	0.01	< 1	0.00
								Time × group	8460	4.66^***^	0.08	3.15^*^	0.05
								Stimt × time × group	16, 920	1.05	0.02	< 1	0.02
								Group	2115	2.01	0.03	1.44	0.03
Skin conductance	Descriptives depicted in Figure response (amplitude)1							Stimtype	2214	9.53^***^	0.08	< 1	0.01
								Stimt × group	4220	1.54	0.03	1.32	0.02
								Group	2110	2.17	0.04	1.64	0.03

*Note:* Effect size *η* ^2^ small (0.01), medium (0.06) and large (0.14).

^#^ *p* < 0.1, *p < 0.05, **p < 0.01, ***p < 0.001.

The second hypothesis was that the AN group would experience more difficulty expressing emotions verbally when presented with emotional pictures. This could not be confirmed, as there was no significant difference in the frequency of emotions words used between the groups. There was a group effect for the use of non‐emotion words, where the AN group used more non‐emotion words than the HC and RC groups (Posthoc group differences AN vs HC *p* < 0.045, AN vs RC *p* < 0.044, HC vs RC ns). Additionally, there was a significant group effect on valence ratings of emotion pictures, with the AN group showing lower positivity overall. The interaction between group and stimulus type showed a trend, not reaching significance. No significant group or interaction effects were found for intensity (arousal) ratings. Interestingly, all group effects disappeared after controlling for depression ratings.

### 3.3 Physiological Measures: Heart Rate and Skin Conductance at Baseline and in Response to Emotion Picture Viewing

**FIGURE 1:**
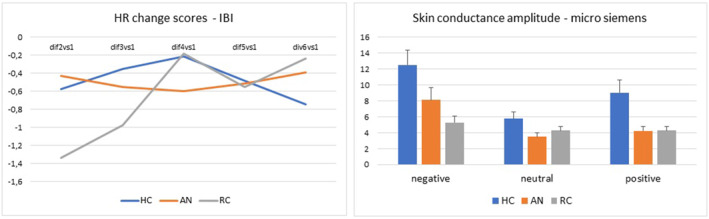
Physiological responses to viewing emotion pictures: Heart rate change pattern from before stimulus presentation & maximum skin conductance amplitude.

Results of the physiological measures are found in Figure 1 and Table 2. The third hypothesis was that AN patients would show signs of physiological hypo‐arousal. The results were mixed. There were no significant group differences in baseline HR measurements. Change in interbeat HR (in reaction to viewing emotional pictures) demonstrated an interaction between time and group. The AN group's response pattern was less responsive (flatter) than those of the HC and RC groups. No other interaction or group effects reached significance. After controlling for the possible confounding effects of depression ratings, the results remained unchanged.

For the skin conductance responses no significant group effect on amplitude was found, nor an interaction between stimulus type and group.

## 4 Discussion

The aim of this study was to gain insight into alexithymia and emotion processing in patients with AN, compared to RC and HC. This was investigated with a comprehensive mix of self‐report, behavioural and physiological measures.

With regard to the first hypothesis, patients with AN reported more alexithymia and difficulties with emotion regulation than HC participants. The RC group performed in between the other two groups on alexithymia, and equally to HC on difficulties with emotion regulation. Overall, the RC group had scores in the healthy range on BMI and all measures of psychopathology.

The second hypothesis could not be confirmed, since there were no group differences in verbal emotion expression when presented with emotion pictures. Depression ratings accounted for group differences in use of non‐emotion expression, suggesting that they are linked to mood and state rather than being specific to anorectic traits.

Analyses with regard to the final hypothesis produced mixed results. Unexpectedly, no differences in baseline heart rate were found, despite bradycardia being common in AN (Peyser et al. 2021). One possible explanation for this is that all patients in the present study were in treatment and at various stages of physical recovery from starvation. The AN group demonstrated a less responsive heart rate pattern when viewing emotion pictures than the recovered group and the control group, with small to medium effect sizes. Contrary to findings that AN is related to lower electrodermal activity (Ralph‐Nearman et al. 2024), group differences in this study did not reach significance. Although a main effect of stimulus type on valence and intensity ratings in the current study hinted at a correct stimulus interpretation by participants, the overall level of physiological arousal caused by viewing the pictures might have been too limited to disentangle group differences.

To conclude, there were only subtle findings of reduced physiological responsiveness in AN that were not found in the RC group.

Overall, a pattern of full recovery from anorexia nervosa is seen in the current RC group, which pleads for more state dependent difficulties in emotion processing than an underlying trait in the AN group.

### 4.1 Methodological Considerations

Overall, it is noteworthy that the results showed no interactions between stimulus type (negative, neutral or positive) and group (AN, RC or HC) in response to viewing emotion pictures for both behavioural and physiological measures. This indicates that the group differences did not vary between intense and neutral emotion pictures, suggesting a subtle, more general dampening of responding rather than a specific lack of response to specific emotions.

Another interesting methodological consideration is that the effect sizes of the group differences in both behavioural and physiological measures were small to medium compared to the large effect sizes observed in the self‐report questionnaires on alexithymia and emotion regulation. AN patients present severe clinical dysfunction in self‐reports which is higher than expected based on the results of the current behavioural and physiological performance.

### 4.2 Alexithymia in AN Different From Other Groups?

The question from the introduction was whether alexithymia works differently in anorexia nervosa than in other diagnostic groups. In the present study, patients diagnosed with AN showed subtle reduced physiological response to general emotional stimuli, whereas the ability to recognise and name emotions remained intact. The original theory of alexithymia would expect the opposite. Nevertheless, in order to reach more definite conclusions, a direct comparison with other diagnostic groups would be needed, for example a group of HC with elevated alexithymia or a group of patients with autism spectrum disorders. It is noteworthy that the expected emotion recognition and expression pattern corresponding to the original theory of alexithymia is not always present in high alexithymia groups (other than patients with AN) as well, as both over‐ and under‐physiological responding have been observed in high alexithymia groups. The most recent theory of alexithymia (Panayiotou et al. 2018) posits that individuals with high alexithymia levels experience reduced flexibility in their responses to stressful stimuli or situations. This pattern may also be applicable in AN, and further research is warranted.

### 4.3 The Recovered Group, Recovery From AN

As AN is a severe condition that often has a chronic course, and emotional functioning is known to play a role in maintaining the disease, it is interesting to learn from the data of recovered patients relating to alexithymia. The following conclusions can be drawn from the present study about the state versus trait characteristics of alexithymia. Controlling for depression as a state variable, leads to disappearing group differences in behavioural findings but not in physiological findings in AN. In general, the RC group showed similar scores to the HC group on all measures (questionnaires, behavioural and physiological) and only showed significant non‐clinical elevated alexithymia scores. In this sample, alexithymia appears to reflect a state‐dependent rather than a trait‐like characteristic. However, definitive conclusions require longitudinal research, as it remains unclear whether the RC participants experienced lower levels of alexithymia during their active phase of the eating disorder compared to the current AN group.

Overall a pattern of full recovery from anorexia nervosa is seen in the current RC group and that pleads for more state dependent difficulties in identifying and describing emotions, the core features of alexithymia, as well as emotion regulation, than an underlying trait in the AN group.

### 4.4 Strengths and Limitations

The strengths of the present study lie in the multiple groups and the extensive mix of measures (self‐reports, behavioural and physiological) used to capture various aspects of alexithymia and emotion processing.

There are also limitations, one is the study design, which might have been too simple in asking to identify very clear non‐ambiguous pictures with emotional meanings. The selection of straightforward visual stimuli, although well‐known for eliciting emotions (Branco et al. 2023), might have been too straightforward to elicit alexithymia in the behavioural measures, due to ceiling effects. At least both behavioural and physiological measures were able to detect the intended directions in stimulus types (i.e., a differential pattern for intensive vs neutral stimuli as found earlier by Lang et al. (1999) and D'Hondt et al. (2010)). Next, it is uncertain that all other possible confounding effects were accounted for and that the findings are specifically related to alexithymia. For example, starvation is known to affect psychological and cardiac functioning (Spettigue et al. 2025) and we do not exactly know what the nutritional status of the subjects was besides BMI. Furthermore, other co‐morbidity than depression ratings might also co‐occur and partly overlap with alexithymia, for example stress related disorders like PTSD are known to impact the biological freeze responses and subjective experience (Roelofs 2017). Another limitation is that even though the sample size of the present study was larger than several other studies that performed experimental research with behavioural and or physiological outcome measures with AN patients, the sample size of the present study might have been too small to detect more subtle effects, especially in complex interactions between three group and multiple task conditions, which leads to a risk of Type 2 errors.

### 4.5 Future Directions

In future studies, it would be interesting to compare different kinds of emotion‐eliciting materials with for example social, eating disorder and general emotion eliciting material, presented in more ecological way for example in a movie or VR setting. It would be interesting to vary complexity of the emotions elicited. This could be done in a more extensive test battery with a more interactive experimental task design to elicit stress as van der Vijgh et al. (2014).

Furthermore, it might be interesting to make a difference between how participants rate and describe their feelings and identify what the correct response to an emotion context should be, to disentangle the experience‐part from knowing the intended meaning of emotions.

Next, it would be interesting to investigate specific effects of nutritional status in relation to starvation on the ability to process emotions to better capture state effects of AN.

Last, it would be very interesting to use a longitudinal design starting measurements pre‐treatment until full recovery to investigate the timing of changes in eating behaviours, weight, physical fitness, depression, alexithymia and emotion regulation on a physical as well as psychological level.

## 5 Conclusion

The objective of this study was to gain more insight into the nature of alexithymia in AN patients and RC participants. Although high levels of negative affect, alexithymia and emotion regulation problems were found with self‐report questionnaires in patients with AN, they showed only subtle signs of general reduced physiological responsiveness when viewing emotion pictures, the AN group was equally able to identify and name basic emotions as the HC group. The effect sizes of the group differences in both behavioural and physiological measures were small to medium compared to the large effect sizes observed in the self‐report questionnaires on alexithymia and emotion regulation. It appears that people with anorexia nervosa perceive themselves as more inadequate than the behavioural and physiological data would justify in the acute phase of the disease. The RC group only showed differences from the HC group in elevated but non‐clinical self‐assessed alexithymia ratings. Therefore alexithymia in AN appears to be more state than trait.

Longitudinal studies are recommended with a task design that can vary in complexity of emotions and with a more broad range of emotion induction to gain more insight into the different parts of the recovery processes at play at different time points.

## Funding

The authors have nothing to report.

## Ethics Statement

The study was approved by the ethics committee of the department of Social Sciences of the Radboud University in Nijmegen (ECSW20171303‐496 and ECSW‐2020‐070).

## Consent

The study was carried out in accordance with the Declaration of Helsinki. Prior to the start of the experiment all subjects gave their written informed consent.

## Conflicts of Interest

The authors declare no conflicts of interest.

## Permission to Reproduce Material From Other Sources

Yes, the IAPS pictures were obtained via Radboud University Nijmegen.

## Data Availability

The authors have not made the data available for general use. Interested researchers can request access.
